# Hallmarks and Biomarkers of Skin Senescence: An Updated Review of Skin Senotherapeutics

**DOI:** 10.3390/antiox12020444

**Published:** 2023-02-10

**Authors:** Darya Bulbiankova, Rocío Díaz-Puertas, Francisco Javier Álvarez-Martínez, María Herranz-López, Enrique Barrajón-Catalán, Vicente Micol

**Affiliations:** 1 Institute of Research, Development and Innovation in Health Biotechnology of Elche (IDiBE), Universitas Miguel Hernández (UMH), 03202 Elche, Spain; 2 Institute of Sanitary and Biomedical Research of Alicante (ISABIAL), 03010 Alicante, Spain; 3 CIBER, Fisiopatología de la Obesidad y la Nutrición, CIBERobn, Instituto de Salud Carlos III (CB12/03/30038), 28029 Madrid, Spain

**Keywords:** aging, biomarker, ROS, SASP, senescence, senotherapy, skin, antioxidant

## Abstract

Aging is a complex process characterized by an ongoing decline in physiological functions, leading to degenerative diseases and an increased probability of death. Cellular senescence has been typically considered as an anti-proliferative process; however, the chronic accumulation of senescent cells contributes to tissue dysfunction and aging. In this review, we discuss some of the most important hallmarks and biomarkers of cellular senescence with a special focus on skin biomarkers, reactive oxygen species (ROS), and senotherapeutic strategies to eliminate or prevent senescence. Although most of them are not exclusive to senescence, the expression of the senescence-associated beta-galactosidase (SA-β-gal) enzyme seems to be the most reliable biomarker for distinguishing senescent cells from those arrested in the cell cycle. The presence of a stable DNA damage response (DDR) and the accumulation of senescence-associated secretory phenotype (SASP) mediators and ROS are the most representative hallmarks for senescence. Senotherapeutics based on natural compounds such as quercetin, naringenin, and apigenin have shown promising results regarding SASP reduction. These compounds seem to prevent the accumulation of senescent cells, most likely through the inhibition of pro-survival signaling pathways. Although studies are still required to verify their short- and long-term effects, these therapies may be an effective strategy for skin aging.

## 1. Introduction

Since the dawn of civilization, humans have been confronted with the problem of aging and mortality and have therefore sought ways to slow this process, if not defeat it altogether (e.g., the philosopher’s stone myth). Currently, the question of delaying aging is as relevant as ever: more than 10% of the world’s population is over 65 years old, and Europe is the “oldest” region, being home to 19% of those over 65 [1]. According to the United Nations forecast report, the total number of people over 60 will double between 2017 and 2050, rising from 962 million to an astounding 2.1 billion [2]. Consequently, our lifespans are rapidly outpacing the so-called health span. Unfortunately, wisdom is not the only gift of maturity; it also bears a heightened risk of geriatric conditions such as frailty, impaired mobility, and cognitive deterioration. Elderly individuals are sick for longer on average, and it is not uncommon for them to struggle with multiple chronic issues simultaneously, placing a sizable burden on the medical system.

In general, aging is viewed as a multifactorial process in which the body changes over time, leading to functional impairment and an ever-increasing likelihood of the loss of life. The problem of aging encompasses a web of aspects, from obvious clinical effects to sociopolitical outcomes for the lives of individuals and society as a whole. These aspects are the subject of the study of gerontology [3]. The subfield of gerontology that deals with the study of aging in vitro by analyzing the mechanisms of aging in cultured cells is called cytogerontology [4]. It should be noted that cytogerontological findings cannot be the sole basis for proposing theories due to scientific reductionism. Most models of normal or altered aging in multicellular organisms are reduced to several specific molecular switches in a set of cells (sometimes limited to a single cell line). As a result, most of these models are devoid of the complexity of neural and humoral influences, which makes them quite vulnerable when translating in vitro data to in vivo models.

In 2013, López-Otín et al. [5] described nine now-canonical hallmarks of cellular and molecular aging and grouped them into three categories. The primary hallmarks include developments at the molecular level: telomere attrition, the instability of the genome, epigenetic alterations, and a loss of proteostasis—all of which have the potential to wreak havoc on the molecular level. Antagonistic hallmarks comprise a counteractive response to the embodiment of the primary hallmarks (deregulated nutrient sensing, mitochondrial dysfunction, and cellular senescence). Finally, integrative hallmarks such as stem cell exhaustion and altered cellular communication are the culmination of damage caused by the aforementioned hallmarks, leading to what is known as a damaged phenotype, i.e., functional deterioration.

While the primary hallmarks, as the name implies, are the starting point of damage, they alone do not constitute evidence of cellular aging. The downstream response is, in a sense, a counterbalance that serves to bring the system to homeostasis after initial detrimental effects. Unfortunately, over time, if left to its own devices, this mechanism can lead to additional damage, which, in turn, causes harm to the body. As a result, physiological and functional damage accumulates, setting off the onslaught of chronic inflammation and disease. Impaired energy metabolism alone can lead to changes in insulin sensitivity that affect a whole range of normal functions, from neural to sensory (especially visual and auditory) [6]. It must be stipulated that cellular senescence and aging are not transposable concepts. Aging is a process that is inherently time-dependent, whereas senescence occurs throughout life, even before birth during embryogenesis.

Recent advances in our understanding of the factors and mechanisms that cause and sustain cellular senescence may lead to the selective removal of senescent cells. The term senotherapy has been used to describe this unique approach [7]. Senotherapeutics have been proven to reduce the quantity of naturally existing senescent human cells in vitro, reverse or prevent senescence hallmarks, and improve physical and cognitive function, lengthening the lifetimes of aged mice [8].

The main objective of this review was to describe cellular senescence as a factor in overall aging, as well as skin aging in particular. Additionally, the most important hallmarks and biomarkers of cellular senescence, with a special focus on skin biomarkers and their relationship with oxygen reactive species (ROS), are discussed. Finally, senotherapeutic strategies with antioxidant compounds, especially plant polyphenols, are also reviewed.

## 2. Search Strategy

**Figure 1:**
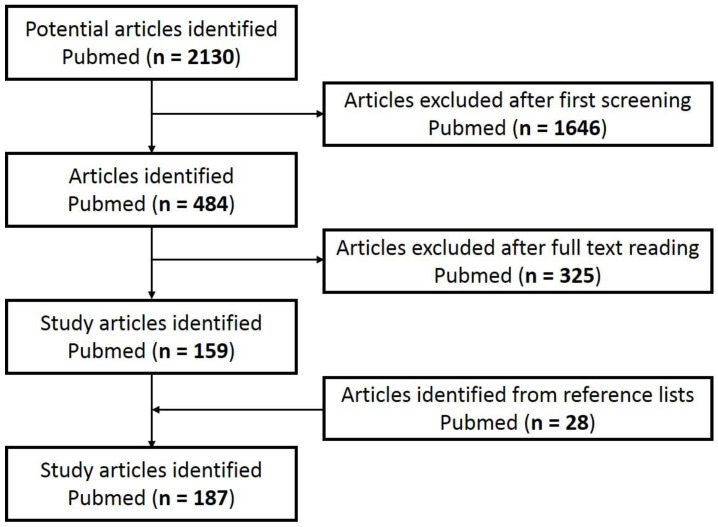
Flowchart of article screening.

A systematic search for hallmarks, biomarkers of skin senescence, and senolytics was performed considering all the articles published until December 2022 through Medline using PubMed as the search engine. Manual research was completed at Miguel Hernandez University (UMH), Spain. The UMH bibliographic resource provided access to original manuscripts. A systematic search was performed using the keywords “cell”, “senescence”, “skin”, “senolytic”, “antioxidant”, “aging”, “biomarker”, “ROS”, “hallmark”, and “senotherapy”, using the operators AND OR between relevant keywords. Eligible abstracts were read to determine if they met the eligibility criteria, which consisted of being as recent as possible (before December 2022), being an original publication belonging to an indexed journal, and being aligned with the objective and theme of the review. Non-English or Spanish language publications were excluded from the present review. Filter limits (such as text availability, article type, and publication date) were not applied. Titles and abstracts that met the inclusion criteria were retrieved, and the full text articles were studied. Finally, 187 studies were selected for review (Figure 1).

## 3. Cellular Senescence

The first description of the phenomenon of cellular senescence was put forth by Hayflick and Moorhead in a formative 1961 paper [9]. In this paper, the authors scrutinized and challenged the previously held view postulating that all cells are essentially immortal and therefore can duplicate infinitely. Hayflick and Moorhead showed that after a series of in vitro passages, normal human fibroblasts enter an irreversible growth arrest that fundamentally differs from the behavior of a typical cancer cell. This occurrence was later termed the Hayflick limit by Macfarlane Burnet and is now commonly known as “replicative senescence” [10]. It has long been suspected that cellular senescence plays a role in perpetuating aging, but it was not until 2011 that this was convincingly demonstrated by the in vivo research of van Deursen et al. [11]. Currently, the term cellular senescence is used to refer to cells shifting into stable cycle arrest, meaning that a normally proliferating cell becomes impervious to division incitement, despite optimal growth conditions and mitogenic stimuli. This phenomenon is typically associated with DNA damage [12].

It must be stipulated that cellular senescence and aging are not transposable concepts. Aging is a process that is inherently time dependent, whereas senescence occurs throughout life, even before birth during embryogenesis. Senescence can be described as a dynamic, multifactorial process in which cells undergo specific changes and transformations depending on their environment. These cells remain viable and metabolically active, albeit with additional alterations to normal function, and are remarkably resistant to apoptosis in most cases [13,14].

**Figure 2:**
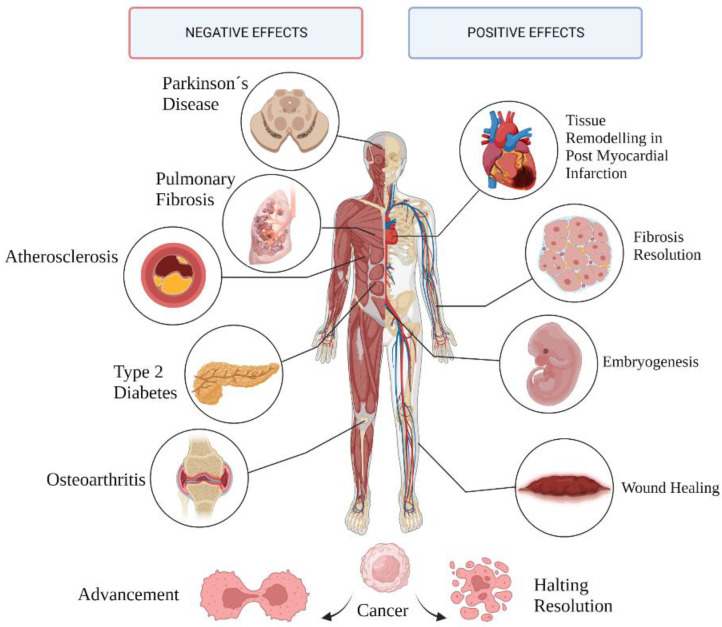
Pathological and physiological outcomes of cell senescence.

Senescence is linked to several normal and pathological mechanisms involving tissue remodeling (Figure 2). While the initial, transient accumulation of senescent cells performs beneficial functions, it is a double-edged sword when persistent, negatively affecting surrounding cells, tissues, and the body as a whole.

The bilateral nature of senescence is most evident with respect to its role in cancer [15]. The underlying mechanism of cell cycle arrest prevents the multiplication of cells with amassed DNA damage, which are later cleared out by immune cells, contributing to overall structural equilibrium [16,17]. Senescent cells tend to accumulate with age, most likely due to the inability of the aging immune system to promptly rid tissues of these cells, usually resulting in permanent damage [18]. Senescent cells can directly affect neighboring cells by secreting senescence-associated secretory phenotype (SASP) factors—an elaborate mixture of pro-inflammatory molecules, namely, cytokines, prostaglandins, miRNAs, and damage-associated molecular pattern proteins (DAMPs); chemokines attracting roving immune cells; and proteases capable of damaging the composition of the extracellular matrix (ECM). This secretory phenotype can create proinflammatory conditions favorable for cancer cell proliferation and expansion [19,20]. Nonetheless, the make-up of secreted SASP varies considerably according to cells and tissues, as well as the stimuli provoking the onset of senescence.

In skin lesions, senescent fibroblasts secrete platelet-derived growth factor AA during the proliferative phase of wound healing, stimulating the differentiation of adjacent fibroblasts into myofibroblasts and thus contributing to the shrinkage of the wound opening, optimizing tissue repair [21]. Furthermore, at the end of the wound healing process, myofibroblasts lose their activity by becoming senescent, which also promotes their elimination by the immune system [22,23,24].

## 4. Other Nonsenescent Forms of Cell Cycle Arrest

Senescence is recognized first and foremost by its primary feature—irreversible cell cycle arrest. According to Blagosklonny, senescent cells enter active arrest at the later stages of the G1, G1/S, and G2 phases of the cell cycle [25]. Cell cycle arrest is not senescence but simply one aspect of the senescence equation. Growth stimulation, the second component, is what truly results in the senescent phenotype. This strengthens the argument that cancer and aging share many characteristics and that the secretory phenotype leads to cancer.

It is extremely important to effectively differentiate senescence from alternative forms, namely, quiescence, terminal differentiation, and T-cell exhaustion (Table 1). Quiescence is a state of a replication-competent cell wherein the cell undergoes proliferative arrest after experiencing signals of mitogen deprivation, contact inhibition, etc. [26]. Terminal differentiation is a genetically preprogrammed developmental process and is characterized by the metamorphosis of a series of undifferentiated progenitor cells into specialized progeny and prolonged or permanent cell cycle arrest [27]. T-cell exhaustion refers to a phenomenon of the functional inadequacy of antigen-specific T cells and, in some cases, even their full-on elimination. T lymphocytes are thought to undergo growth arrest after prolonged exposure to viral and tumor cells, and even after their elimination, T cells remain “wired” to stay in the exhausted state, especially in cases of secondary activation upon reinfection [28].

## 5. Hallmarks of Senescence

The hallmarks of senescence are a set of characteristics or alterations acquired by cells as they transition to senescent states, e.g., morphological changes. A “biomarker” (an amalgamation of “biological marker”), in its broadest sense, is a quantifiable measure of a certain biological parameter or condition that can be measured with a certain degree of accuracy and reproducibility. Biomarkers are often associated with hallmarks, e.g., wide and flattened cells, which are framed within morphological changes. Stephen Naylor defined a biomarker as “an umbrella coalescence term” and, more precisely, “a characteristic that is objectively measured and evaluated as an indicator of normal biological or pathogenic processes” [50]. Regarding the interpretation of biomarkers of aging, one of the first definitions was proposed by Baker et al. in 1988: a “biomarker of aging is a biological parameter of an organism that either alone or in some multivariate composite will, in the absence of disease, better predict functional capability at some late age, than will chronological age” [51].

In the following sections, several standard in vitro hallmarks and their associated biomarkers for senescence detection and their prospective applications are described.

### 5.1. Stable Cell Cycle Arrest

**Figure 3:**
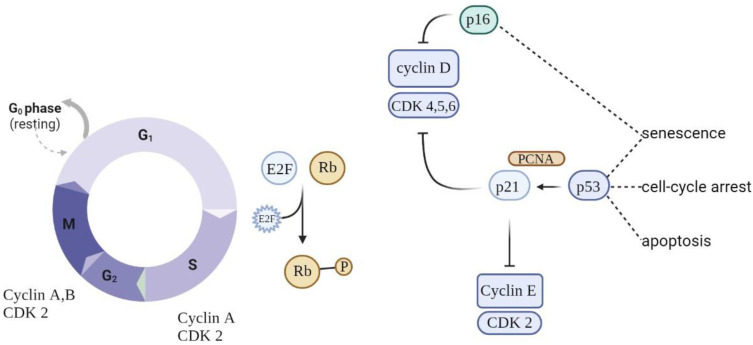
Schematic of cell cycle regulation (partial). To progress through the G1 phase, cyclin and CDKs should be set up and further phosphorylated. CDK inhibitors (CDKIs) halt cyclins from acting as cell cycle breaks. A close connection between cyclins, CDKs, and CDKIs is essential for correct cell cycle progression.

Eukaryotic cell division is directed by a group of heteromeric serine/threonine kinases known as cyclin-dependent kinases (CDKs) [52]. The basic mode of action is the consecutive activation of each member of the family network, bringing about the phosphorylation of appropriate substrates and thus the passage through each step of the cell cycle (Figure 3).

Cell cycle arrest is prompted by the p53/p21^WAF1/CIP1^ and p16^INK4A^/pRB tumor suppressor routes. p21^WAF1/CIP1^ acts downstream of p53, while p16^INK4A^ acts upstream of pRB. Prolonged stress conditions may be a potential trigger for the activation of p16^INK4a^—a cell cycle regulator that acts as an inhibitor of CDK4/6 kinases [53]. This leads to arrest in the phosphorylation of RB, which in turn contributes to enduring cell cycle arrest. As long as p16^INK4a^ is expressed, Rb proteins remain in a sustained hypophosphorylated state, stimulating attachment to E2F and cell cycle exit in G1 [54]. The overexpression of p16^INK4a^ was detected in aging human skin, indicating a possible link between this suppressor protein and aging and senescence. Moreover, the upregulation of p16^INK4a^ was observed in senescent fibroblasts in response to oxidative and DNA-damaging stressors.

When p53 is switched on, it acts as a regulator of the growth-suppressive transcriptional process by activating the cyclin-dependent kinase inhibitor gene p21, indirectly causing the hypophosphorylation of RB and cell cycle arrest [55,56]. Nevertheless, these genes are not absolute markers: the persistent activation of p21^CIP1^ (relevant in the launching of the senescence process) is not always observed in senescent cells, making it unreliable as a single marker [57]. At the same time, p53 is a regulator of apoptotic cell death, making it a dubious aid in discriminating between senescent cells and those undergoing apoptosis [58].

### 5.2. Metabolic Changes

In 1995, Dimri et al. detected the senescence-associated β-galactosidase (SA-β-gal) enzyme with optimum levels at pH 6 expressed by senescent cells; since then, it has become the best-known and most studied biomarker of cell senescence [59]. As cells age, their defense mechanisms become further corrupted, leading to amassed molecular debris [60], often associated with the altered function of lysosomal and proteosomal enzymes [61]. Detecting switches in normal metabolic function is a useful way to identify senescent cells. An increase in SA-β-gal is a biomarker that reflects the increase in the number and size of lysosomes [40]. SA-β-gal is not detected in quiescent or differentiated cells [40], yet it has been found in cells with intrinsically high lysosomal β-galactosidase activity, such as macrophages and postmitotic cells [62], and in several types of cancer cells [63]. Hence, the choice of SA-β-gal as an exclusive marker may lead to a false-positive outcome and requires the amplification of results using auxiliary markers.

A more recent enzymatic marker for senescence-associated lysosomal expansion, α-fucosidase, was found to be overexpressed in multiple senescent cell models [64]. Additionally, the attenuation of α-fucosidase was established to negatively affect the onset of senescence [65].

The enlargement of lysosomal substances in senescent cells is largely attributed to the accumulation of lipofuscin (LF) [66]. LF is represented by nondegradable yellowish-brown pigment granules that are mainly composed of an autofluorescent mixture of oxidized lipids, cross-linked proteins, oligosaccharides, and metals. This aggregate is a derivative of the oxidative and polymerization reactions between a wide range of cellular structures and macromolecules [67]. Because of its convoluted chemical structure, LF cannot be eliminated, causing it to accumulate in the lysosomes or cellular cytoplasm of lingering postmitotic and senescent cells that remain after normal autophagy. Over time, this leads to the extension of the lysosomal lumen to accommodate the ever-increasing amounts of LF. In contrast, proliferation-competent cells systematically attenuate the volume of LF during cell division [68]. For this reason, LF is commonly linked to aging and is referred to as the “age pigment” [69]. LF can be detected by microscopy techniques such as transmission electron [66] or confocal fluorescence microscopy [68].

Generally, metabolic changes in cell senescence are denoted by an increase in the ratio of AMP/ADP to ATP, leading to the activation of AMP-activated protein kinase (AMPK) signaling. The upregulation of AMPK causes a shift from a biosynthetically driven metabolism into a catabolism mode. Aging-related diseases are greatly impacted by the ability of AMPK activation to slow or stop the aging process. By the same token, in skin, senescence has been shown to affect various metabolic pathways in both dermal and epidermal cells, leading to deficiencies in key metabolites and protective proteins, which weakens the skin’s barrier function [70].

As a byproduct of mitochondrial metabolism, ROS are continuously created and removed by antioxidant mechanisms. To function properly, respond to metabolic stress, and avoid cellular senescence, ROS produced by mitochondria must be controlled. Mitochondrial ROS are a physiological activator of AMPK, and this activation results in an antioxidant response, reducing the amount of mitochondrial ROS produced. However, AMPK-deficient cells exhibit elevated amounts of mitochondrial ROS and develop premature senescence. These findings accentuate the critical role of AMPK in detecting and neutralizing mitochondrial ROS to maintain cellular metabolic equilibrium and resilience to stress and senescence [71].

### 5.3. Morphological Changes

During the transition to the senescent state, cells undergo a few changes in their morphological features [72]. The most obvious are the atypically distended dimensions and flat shape [73]. Cells exhibit substantial vacuolation and in some cases have multiple nuclei [74]. Moreover, adherent cells appear flattened, disarranged, and show random orientation in the culture plate [75].

Senescent cells often exhibit an augmented nucleus [76], which has been attributed to a range of factors, including a reduction in lamin B1. Lamin B1 is an intermediate filament protein incorporated into the inner portion of the nuclear envelope [77]. It aids in the stability of the nucleus, the replication of DNA, gene transcription, and cell proliferation. A lamin B1 deficit is part of the senescence-induced restructuring of the nuclear architecture. It is accompanied by the refashioning of chromatin organization, the loosening of heterochromatin, and the resulting enlargement of the nucleus [78,79].

Senescent cells exhibit a substantial increase in the number of mitochondria, and these organelles present an enlarged, distended shape. The enlargement of mitochondria can be attributed to the cell compensating for dysfunction by fusion and reduced division. Due to reduced autophagy in senescent cells, poorly functioning mitochondria are not cleared but rather accumulate in the cell. This amassing leads to a decrease in the mitochondrial membrane potential, accelerating the production of ROS. In addition, it has been observed that ROS have a damaging effect on mitochondrial DNA, which exacerbates organelle dysfunction. This is particularly evident in cases of photoaged skin, where mitochondrial DNA has a high level of mutations due to exposure to UVA compared to protected skin [80].

### 5.4. Epigenetic Alterations

Chromatin reorganization is a prominent genome-wide alteration in cells experiencing senescent transformation, which contributes to persistent proliferation arrest and transition into full senescence [81]. Senescent cells exhibit regions of condensed heterochromatin observed under microscopy [82]. These regions, known as senescence-associated heterochromatin foci (SAHF), are densely organized patches of DNA containing a constitutive heterochromatin marker histone H3 (H3K9me3—trimethylated at Lys9) in the core surrounded by the facultative heterochromatin marker H3K27me3 (trimethylated at Lys27).

Constitutive heterochromatin proteins, namely, di- or trimethylated forms of histone H3 (H3K9me2/3) and HP1 proteins associated with gene silencing, are easily detectable by immunofluorescent techniques, which highlight their role as potential senescence markers [83]. Notably, SAHFs do not carry sites of active transcription; thus, SAHFs are true transcriptionally inactive patches of heterochromatin. SAHFs are routinely identified by staining DNA with specific dyes such as DAPI, with senescent cells exhibiting spotty staining and “normal” nonsenescent DNA showing uniform staining [84].

Altered redox mechanisms have been observed to cause the general hypomethylation of the genome and the specific hypermethylation of DNA promoters, although it is not clear whether ROS-induced changes in the epigenetic makeup are exclusively a cause or a consequence of aging. Broad-based DNA hypomethylation has been shown to be strongly implicated in the expression process of aging genes, which is supported by the fact that cancer, a high-risk age-associated disease, shows the most pronounced effects of ROS-dependent DNA methylation. In aging, genomic regions with the activating histone post-translational modification H3K4me1 are more likely to have hypomethylated DNA sequences. The duality of the hyper- and hypomethylation processes of the different parts of the DNA reveals the complexity of the genomic mechanisms involved in cell senescence [85]. Nevertheless, aberrant DNA methylation could be both a potential biomarker and a tool to evaluate therapeutic treatments.

### 5.5. DNA Damage and Persistent DNA Damage Response

DNA damage, especially double-strand breaks (DSBs), is an integral aspect of senescent cells. DNA damage response in senescent cells is most often associated with proteins such as γH2AX (phosphorylated at Ser139) and an adjacent p53-binding protein 1 (53BP1), which are known to aggregate at DSBs. The breaking of the double strand activates the recruitment pathway of the ataxia-telangiectasia mutated (ATM) and ataxia-telangiectasia and Rad3-related (ATR) protein kinases to the site of damage. These kinases are capable of converting histone H2AX to its phosphorylated form γH2AX, which quantitatively correlates with DSBs. ATM is known to phosphorylate a variety of substrates, particularly the checkpoint kinases CHK1/2, facilitating a downstream phosphorylation cascade and the activation of the p53/p21 signaling pathway [86]. The simultaneous detection of γH2AX and p53/p21 could be a viable option for detecting senescent cells.

Cellular senescence is also marked by the elevated expression of the promyelocytic leukemia protein (PML) [87]. PML is a normal constituent of the nucleus of most cell lines and acts as part of cell cycle regulation through the Rb and p53 pathways, which tend to accumulate in PML nuclear bodies. The extent to which PML accumulates in regions of unprocessed, unrepaired DNA correlates positively with the extent of DNA damage signaling, indicating that the transition to the senescent state may be associated with the corruption of the cell’s DNA repair system [88].

Another noteworthy epigenetic change connected to senescence is the formation of so-called DNA-SCARS (DNA segments with chromatin alterations reinforcing senescence). These foci appear to be a universal feature of most types of senescence, showing coupling with PML core bodies as well as an accumulation of activated forms of p53, ATR, and ATM [89,90]. Thus, these persistent foci could be considered excellent senescence comarkers.

Telomere dysfunction-induced foci (TIF) are an alternative version of DNA-SCARS, specifically located at uncapped telomere sites. These markers have been shown to concentrate in both senescent cells and aging tissues, as determined by the colocalization of 53BP1 and γH2AX at the telomeric ends of DNA [91]. Studies have associated a longer telomere length with decreased cellular senescence. Mice with hyper-long telomeres have been shown to express lower levels of global DNA damage, telomere-induced DNA damage, and p21, revealing the relationship between cellular senescence and telomeric length [92].

### 5.6. Apoptosis Resistance

Senescent cells employ a number of pathways to evade apoptosis [93]. Major survival pathways include ephrins and the Bcl-2 protein family, which act by actively suppressing apoptosis. Exemplary research conducted on murine models that studied the inhibition of antiapoptotic Bcl-2, Bcl-W, and Bcl-xL proteins showed apoptosis and the subsequent elimination of senescent cells [94]. Escape from apoptosis could also be achieved by the overexpression of p21, which appears to be a substantial inhibitor of p53-dependent apoptosis. Additionally, higher levels of p21 impede the activation of the c-Jun amino-terminal kinase (JNK) and caspase networks, both of which have been implicated in the apoptosis process. The dentification of these Bcl-2 proteins is deemed to be a convenient technique for localizing senescent cells. However, the upregulation of the synthesis of these markers is not limited to senescent cells; blood cells also show the overexpression of anti-apoptotic regulators [95]. While these proteins seem appealing as markers that are usually targets for senolytic agents, their expression is rarely chosen to assess this cell state [96]. Rather, evidence of the absence of annexin V and cleaved caspase-3 is regularly used as a marker to rule out apoptosis as a stress feedback process.

### 5.7. Secretory Phenotype

**Figure 4:**
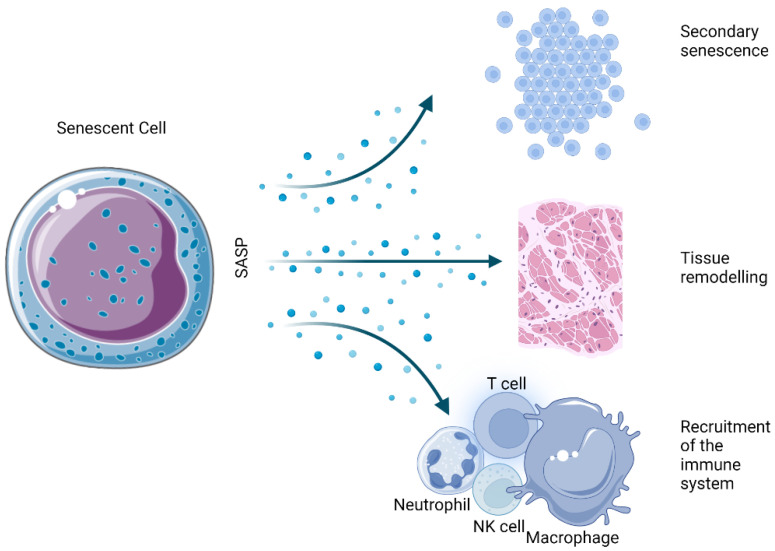
Physiological effects derived from the SASP of senescent cells.

The SASP is one of the most thoroughly described hallmarks of the senescent state. Essentially, it is the ability of senescent cells to send inflammatory signals to neighboring cells in a paracrine manner (Figure 4). Among the several dozen identified factors that are secreted in a cell- and tissue-dependent manner, a number of marker molecules are commonly expressed by most senescent cells.

The chemical composition of the SASP is highly dependent on the type and strength of the stimulus triggering senescence as well as the type of cells implicated in the process. For example, oncogene-induced senescence shows the exaggerated secretion of typical proteins in comparison to replicative or irradiation-induced senescence [97]. Moreover, when examining tissue and tumor material for SASP influences, it is very important to consider that the immune cell infiltrate and the degree of senescent cell accumulation could present additional unpredictable variables [98].

Even so, some crisscrossing has been demonstrated between a set of SASPs, with several proteins detected more or less universally. A basic SASP series consists of soluble molecules such as growth factors (IGFBPs, VEGFs, PDGFs, and HGFs) and interleukins [99]. The leading cytokine in the SASP process is proinflammatory IL-6, which appears to be directly driven by sustained DNA damage in keratinocytes, melanocytes, and epithelial cells, among others [100]. Another notable interleukin upregulated by senescent cells is IL-1, with both IL-1α and IL-1β overexpressed by various cell types [101].

In addition to the secretion of some proinflammatory factors, senescent cells also express enzymes for ECM remodeling, such as matrix metalloproteinases (MMPs), especially MMP1/3/9, which are involved in the breakdown of matrix proteins, serine/cysteine proteinase inhibitors (SERPINs), and tissue inhibitors of metalloproteinases (TIMPs) [45,102].

SASP-associated proteases greatly influence cell homeostasis by solubilizing membrane-associated proteins, breaking up and subsequently degenerating signaling molecules, processing, remodeling, or degrading the ECM [103]. These activities are responsible for the high potency of senescent cells in altering surrounding tissues.

SASPs have long been shown to be promising markers of cellular senescence. Furthermore, SASPs can be qualified and quantified both directly and indirectly by observing their known effects on surrounding cells. Nevertheless, it is important to understand that there are some major limitations inherent to secretory markers. SASPs can vary between cell types and different stages of senescence, and there is a gap in technology that does not allow for the high-throughput analysis of single-cell secretory phenotypes, which would be necessary to isolate a population on this basis [104].

### 5.8. Reactive Oxygen Species

ROS are well-known mediators of the senescence process. The generation of hydrogen peroxide, superoxide anions, and hydroxyl radicals disrupts normal cellular processes, induces senescence, and even leads to cell death [105]. Although the fact that ROS-induced DNA strand damage favors the onset and maintenance of senescence [106] has long been observed, the focus has currently shifted to the role of ROS as a signaling molecule in senescence induction. Signaling pathways closely involved in the senescence process, such as p53, p21, and p16, have been known to prompt ROS production [107], consequently promoting the upregulation of SASP factors [108]. Underscoring this role, McCarthy et al. 2013 showed that antioxidants and low oxygen tension restricted the production of IL-1α and downstream IL-6 and IL-8 [109].

Increasing inflammatory conditions accelerate the production of ROS in mitochondria through the recruitment of the cytokines TNF-α and IFN-γ [110]. Moreover, oxidative stress inhibits sirtuin activity, leading to higher levels of inflammation by inhibiting the superoxide dismutase enzyme (SOD) and preventing the inhibition of proinflammatory cytokines [111].

**Figure 5:**
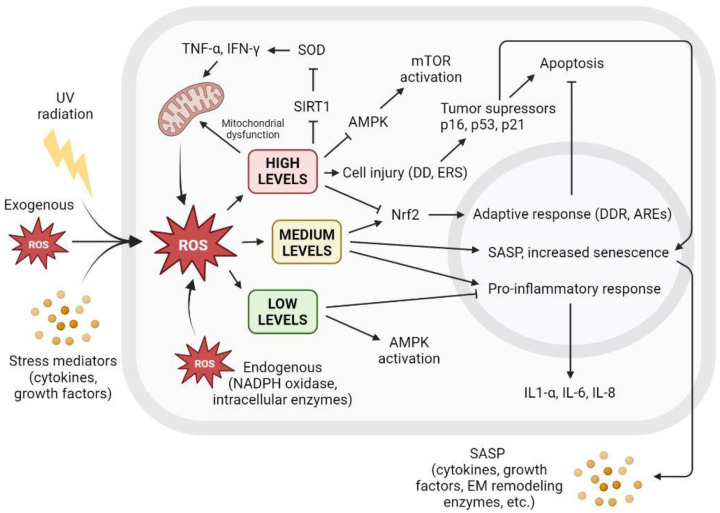
The level of intracellular ROS triggers adaptive response, apoptosis, or SASP. Intracellular ROS levels can vary depending on several factors, including extracellular factors such as exposure to UV radiation, high levels of oxidative stress, or proinflammatory environments with the presence of cytokines or growth factors. ROS are also produced intracellularly as part of normal cellular processes or at elevated levels derived from dysfunctional mitochondrial activity, as well as increased by NADPH oxidase activity or other intracellular enzymes. Low ROS levels activate AMPK and inhibit proinflammatory response. Low ROS levels do not promote cell senescence and contribute to redox homeostasis. Medium ROS levels activate Nrf2 and promote adaptive cellular response to fight ROS and prevent apoptosis, activate proinflammatory response, and increase SASP and its paracrine signaling, promoting cell senescence. High ROS levels cause mitochondrial dysfunctions that generate more ROS; inhibit AMPK, resulting in mTOR activation; and cause widespread cell damage by increasing cellular stress and the concentration of tumor suppressors and cell cycle arrest proteins, leading to apoptosis. AREs: AU-rich elements; AMPK: AMP-activated protein kinase; DD: DNA damage; DDR: DNA damage response; EM: extracellular matrix; ERS: endoplasmic reticulum stress; IFN-γ: interferon gamma; IL: interleukin; mTOR: mammalian target of rapamycin; NADPH: nicotinamide adenine dinucleotide phosphate; ROS: reactive oxygen species; SIRT1: sirtuin 1; SOD: superoxide dismutase; TNF-α: tumor necrosis factor alpha; UV: ultraviolet.

There is a clear relationship between cellular ROS levels and senescence. Low ROS levels are important for maintaining redox homeostasis and adequate antioxidant response. However, the increased accumulation of ROS triggers a variety of cellular responses, leading to irreversible cell cycle arrest that may turn into SASP, affecting surrounding tissue or apoptosis and subsequent cell death. Figure 5 shows the different cellular interactions related to senescence depending on the different intracellular levels of ROS.

Inflammaging was introduced and extensively studied by C. Franceschi, who hypothesized that aging might be connected with an overall chronic increase in mediators of inflammation of a diverse nature [12]. This is presumably due to prolonged exposure to harmful substances during the lifespan or to fluctuations in the gut microbiota and other metabolic disturbances. Inflammaging is an ever-changing process that is easily transmitted by soluble factors to neighboring cells or even systemically [112]. Additionally, inflammaging leads to the chronic activation of the local immune system, resulting in persistent low-grade inflammation, creating a positive response loop with the immune system that affects its normal function. The aging process leads to noticeable changes in immune cells, such as the altered expression of surface markers, the weakening of the protective capacity, and a shift in the balance to the side of proinflammatory cytokine secretion. This acquired phenotype, referred to as “immunosenescence”, adds to the build-up of molecular damage in tissues; exacerbates various conditions; and (notably) significantly reduces the efficiency of the protective response against infection, tumors, and other damage [113].

In chronic cutaneous inflammation, the abnormal accumulation of molecular mediators has been associated with age-related changes in macrophage function [114]. The ability of these cells to carry out surveillance and clearance in an aging body becomes increasingly impaired, which is a key feature of immunosenescence. Subsequently, this “dereliction of duties” leads to an increase in overall oxidative stress and the further promotion of inflammation in the skin milieu.

### 5.9. Biomarkers of Cellular Senescence

To fully appreciate and reap the benefits of senescent cell clearance, it is critical to not only identify a valid marker but also choose a suitable detection method for the identification of said marker. Due to the general ambiguity of the concept of senescence and the diversity of its types, there is currently no universal marker that can selectively identify senescent cells in different tissues and extracellular environments. In the review by Gorgoulis et al., the authors recommended an integrative method consisting of a combination of markers for metabolic changes, nuclear markers, and SASP and/or cell type-specific markers to restrict the allowable margin of freedom and increase specificity [36]. Some of the markers of cellular senescence and the most widely applied methods of their detection are presented in Table 2.

According to the literature analyzed in this review, the most reliable biomarker for determining that a cell is senescent may be the expression of the senescence-associated beta-galactosidase (SA-β-gal) enzyme. This enzyme is a biomarker that can be used to identify senescent cells both in vitro and in vivo. Other biomarkers that tend to persist in senescent cells are the accumulation of DNA damage-associated proteins, such as p16, p21, and p53, and the upregulation of cell cycle inhibitors such as p16 and p21.

Regarding senescence hallmarks, the presence of a stable DDR appears to be the most specific to the presence of senescent cells. This hallmark is characterized by the presence of senescence-associated heterochromatin foci and the upregulation of cell cycle inhibitors such as p16 and p21. Other hallmarks of senescence include the accumulation of SASP mediators, changes in cell morphology and cytoskeletal organization, and increased levels of ROS. However, the presence of DDR can be considered as the most reliable hallmark of senescence.

## 6. Hallmarks and Biomarkers of Skin Aging

There are various crucial factors in the study of skin aging. All skin functions gradually diminish with age [148]. Over time, the skin becomes less elastic and more susceptible to environmental assaults, a circumstance that explains, among other things, the malfunction of the skin as a barrier. Wrinkling, melasma, erroneous wound healing processes, graying, and partial or total hair loss are the most visible signs of older age. Vital functions such as thermoregulation, immunological and nervous skin responses, and cutaneous vascular responses are disrupted. The skin is no longer able to maintain stable energy production, limiting the amount of lipids available, thus becoming increasingly dry [149,150]. Intrinsic skin aging reflects the underlying body deterioration, making it the closest to perfect and most accessible ethical model to study the aging of other tissues arising from the ectoderm, mainly the nervous system. Finally, the prevention and treatment of age-related skin conditions necessitates in-depth knowledge of the skin-aging mechanisms.

**Figure 6:**
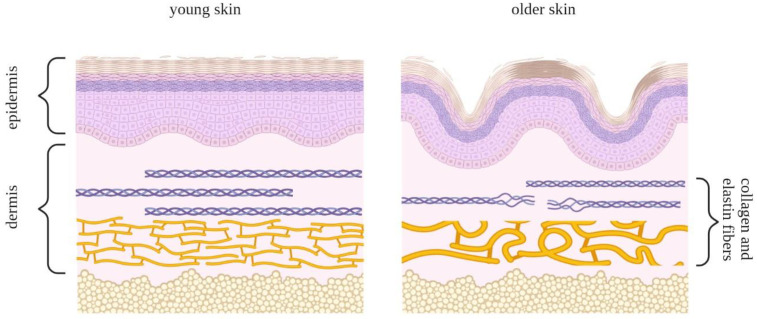
Schematic comparison of young skin (**left panel**) and skin undergoing aging transformation (**right panel**). The epidermal layer comprises keratinocyte cells and consists of five sections: the horny layer, stratum lucidum, granular layer, spinous layer, and basal layer. Collagen and elastin fibers lie underneath these layers in the dermis. These fibers play a role in the rigidity and elasticity of the skin and, with time, are subject to deterioration, leading to the loss of structural uniformity in the skin.

Skin aging is a highly complex process that has a negative effect on most features of normal skin morphology and function and is driven by both intrinsic and extrinsic factors. The intrinsic factors propelling skin aging include the natural passage of time, hormonal regulation, genetic predisposition, and gradual shifts in the cellular redox environment, while the extrinsic factors include environmental stresses such as extensive sunlight exposure and various kinds of pollution [151]. The thinning of the epidermis can be explained to a certain degree by the insufficient proliferative and restorative capacities of the basal epidermal layer as well as by a decrease in the pool of local stem cells. In addition to the epidermis, the dermal epidermal interface and dermis also become thinner (Figure 6) and less vascularized, limiting the access to nutrients and compromising skin homeostasis. Dermal fibroblasts secrete ECM elements, which are important for the structural cohesion and elasticity of the skin. Structural changes in and the degradation of the ECM occur throughout aging, presumably leading to the thinning of the skin, increased wrinkling, and diminishing skin resilience [152].

Extrinsic factors such as excessive sun exposure lead to the skin appearing thick, rough, and almost leather-like, with large broad wrinkles, telangiectasia, and melasma. An important histologic feature of photodamaged skin is solar elastosis (amorphous elastic fiber aggregates) grouped with fragments of poorly organized collagen. This may be the result of the compromised production of elastic fibers and fibrillin, increased degradation by senescence-associated MMPs, or a direct result of UV irradiation [153].

**Figure 7:**
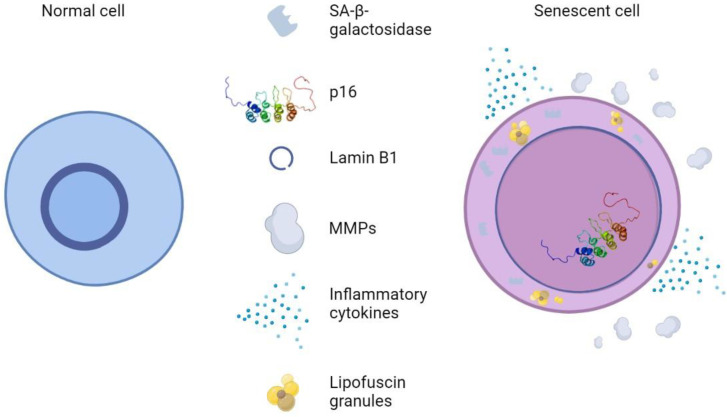
Normal skin cells vs. Senescent skin cells. Senescent cells are distinguished by changes in morphology, such as flattening and increased cell size, pronounced SA-β-galactosidase activity, p16 upregulation, reduction in nuclear lamin B1, and SASP-like MMPs and inflammatory cytokines.

In vitro, skin cells subjected to UV irradiation showed signs of DNA damage and exit from the cell cycle and expressed some of the biomarkers of senescence presented in Figure 7. These include SA-β-gal activity, the enhanced expression of P16^INK4a^, the loss of lamin B1, the expression of MMPs, the secretion of inflammatory cytokines, and the accumulation of LF [154]. UV irradiation also causes the generation of ROS, which maximizes the harmful effects mentioned above, as well as the activation of cell surface receptors. This results in the activation of MAP-kinase p38, JNK, and extracellular signal-regulated kinase (ERK), as well as the recruitment of c-Fos and c-Jun. As a result, MMP1, 3, and 9 are expressed in fibroblasts and keratinocytes via transcription factor activator protein 1 (AP-1). ECM breakdown is accelerated by MMP expression, which is mediated by AP-1. This process is accelerated by the generation of ROS, which also activates MAP kinases and causes the expression of NF-B [155].

Interestingly, the intrinsic aging process plays a role in the change in skin pigmentation. With old age, the number of melanocytes decreases, causing the skin in sun-protected areas to be paler than normal. In contrast, exposed skin acquires uneven pigmentation, with patches of hyperpigmentation [156]. “Solar lentigo” is a common skin condition that usually occurs in the areas most frequently exposed to the sun, especially on the back of the hand, arms, shoulders, and face. This atypical pigmentation is attributed to several processes, such as the hyperactivation of melanocytes, changes in the distribution of the pigment, and the accumulation of LF. Moreover, compromised autophagy in the senescent epidermis could lead to the retention of melanosomes, adding to hyperpigmentation spots [157].

## 7. Skin Senotherapy and Antioxidant Compounds

Senotherapy is an ultranovel segment of anti-aging therapy that addresses approaches to eliminate or even prevent senescence in cells. Although this topic is of great interest, with a sizable number of active trials in humans and even more in the pipeline, there are no drugs officially clinically approved for patient use, which can be attributed to a high standard of current medical interventions as well as the slow-paced nature of preclinical and clinical studies.

Due to an imbalance between pro-oxidant stimuli and antioxidant defenses, oxidative stress can cause cell senescence. It is therefore of great interest to find and characterize antioxidant substances that can prevent or reverse the senescent phenotype [158].

**Figure 8:**
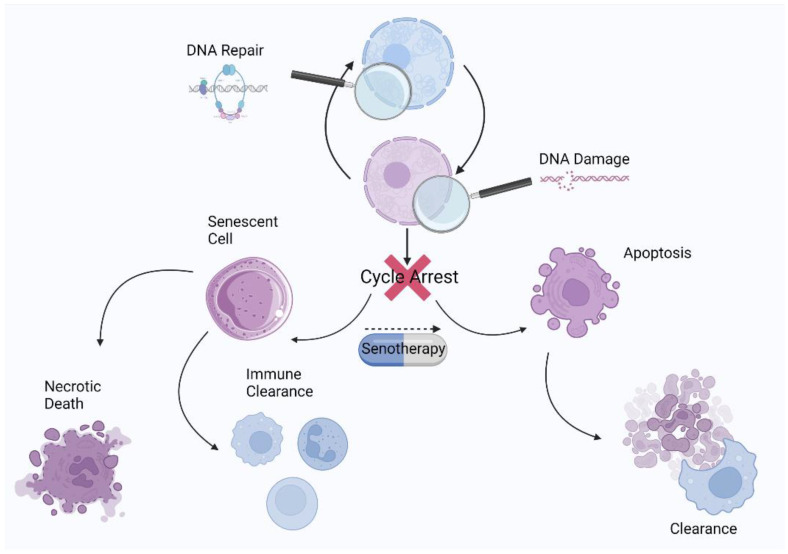
Schematic representation of the cell state after exit from the cell cycle and the role of senotherapy in senescence resolution.

Generally, senotherapy strategies are divided into two groups: senolytics, which act by directly eliminating senescent cells through apoptosis (Figure 8), and senomorphics, which help circumvent the deleterious effects of senescent cells through selective SASP suppression. Unfortunately, senomorphics have a major drawback: unlike senolytics, which exert a cytotoxic effect on senescent cells and can be administered intermittently, senomorphics must be taken regularly to achieve maximum benefit [159], and further studies are needed to determine the efficacy of senomorphics. A growing body of evidence based on preclinical studies in murine models has shown that the periodic purging of senescent cells may be a way to circumvent their persistent deleterious effects while benefiting from their short-term favorable functions [160,161]. The results show that various age-related ailments in geriatric mice can be ameliorated or reversed by this method [8,11]. Certain compounds are currently being tested in human clinical trials for the treatment of geriatric diseases. The compounds with the greatest potential appear to be natural products and/or already approved drugs [162].

A recent study by Boccardi and Mecocci (2021) suggested that natural senotherapeutic agents may be less harmful and more beneficial for humans [163]. Among the natural compounds with senotherapeutic activity, polyphenols stand out, especially flavonoids [164]. Polyphenols are a vast class of plant-derived metabolites that include flavonoids, phenolic acids, lignans, and stilbenes and have been shown to possess multiple biological activities. Among these bioactivities, antioxidant capacity is tightly linked to senotherapeutic activity through ROS scavenging and other oxidative-stress-related mechanisms, such as antioxidant enzyme upregulation [165]. It should be noted that certain polyphenols have antioxidant activity at low concentrations and prooxidant activity at high concentrations [166]. This duality may be useful to promote ROS elimination mechanisms and senescence prevention at low concentrations or enhance its pro-oxidant behavior to trigger selective cell senescence routes and eliminate these cells [158].

Plants rich in polyphenolic compounds have been used in topical and nutraceutical treatments to reverse or halt the skin aging process for a considerable amount of time [167]. Many studies have been conducted on both senescent skin cells and SIPS models, and treatment with polyphenols showed discernible senotherapeutic effects that could be used to treat skin aging and associated conditions. The polyphenols with skin senotherapeutic activity related to their antioxidant capacity are listed in Table 3.

An analysis of the studies compiled in Table 3 found that most of the experiments (64.7%) were carried out in in vitro cell models, while 29.4% were carried out with experimental animals (mice in all cases), and a single experiment was performed on human skin explants. The most common method to accelerate skin senescence and generate a study model is the application of UVA or UVB radiation, although studies have also used other elements such as hydrogen peroxide or prolonged cell growth by counting population doubling steps. The main mechanisms of action of antioxidant senotherapeutic polyphenols for stopping or preventing skin senescence are the scavenging of ROS and the upregulation or activation of antioxidant enzymes, a decrease in MMPs (mainly MMP-1), a decrease in pro-inflammatory ILs (mainly IL-1β and IL-6), a decrease in MAPK, and a decrease in cyclin-dependent kinase inhibitors. The biological effects of these compounds are varied and largely depend on the nature and study model used. The most common effects observed are a decrease in SASP, an increase in collagen production, augmented cell viability, and a slowdown in senescence.

In addition to pure phytochemicals, there exist skin senotherapeutics consisting of plant extracts containing mixtures of phytochemicals that may include compounds from the terpene, alkaloid, and polyphenol families, among others. Rosemary extract, rich in diterpenes and flavanones, has shown antioxidant, photoprotective, and genoprotective activity in human keratinocytes exposed to UVB [182]. Other examples include lemon balm extract, which prevented UVB-induced oxidative stress and DNA damage in human keratinocytes [183], and sweet cherry stem extract, which showed activity against skin-aging-related enzymes, antioxidant capacity, and lipid peroxidation reduction [184]. There is also evidence of synergistic senotherapeutic activity between synthetic antineoplastic drugs and polyphenols. Dasatinib, sold under the brand name Sprycel, entered clinical practice in 2006 and has a reliable safety profile [185]. Following FDA approval, initial clinical trials involving fisetin and a dasatinib/quercetin (D/Q) cocktail have begun, and many more are in progress [161,186,187]. Dasatinib is a tyrosine kinase inhibitor and is used as a drug for the targeted therapy of leukemia. It induces apoptosis in senescent cells through its inhibitory effect on Src tyrosine kinase. The combination of dasatinib and quercetin selectively destroys senescent cells associated with numerous geriatric chronic diseases [188].

In an illustrious clinical trial by Hickson et al. (2019) [186], the D/Q cocktail decreased the number of p16^INK4a^-abundant cells, reduced cells with high SA-β-gal activity, and lowered the concentration of major circulating SASPs. In this Phase 1 pilot study, the D/Q combination was administered in intervals, which the authors referred to as “hit-and-run” treatment. The reason for the chosen method of administration was that dasatinib, as a potent antileukemia drug, has several undesirable side effects that can be avoided by intermittent administration without compromising the senolytic effect [189,190]. Any prospective off-target effects are evaded through sustained receptor occupancy or metabolic pathway modulation.

The most common mechanism of action for polyphenols with senotherapeutic activity is the inhibition of pro-survival signaling pathways. These pathways, such as the phosphatidylinositol 3-kinase (PI3K)/AKT and the MAPK pathways, are activated in senescent cells and contribute to the maintenance of a senescent phenotype. Plant polyphenols have been shown to inhibit these pathways, leading to the induction of apoptosis and the reduction of senescent cell numbers. Another mechanism of action for polyphenols with senotherapeutic activity is the activation of the autophagy process. Autophagy is a cellular degradation process that helps to remove damaged and unnecessary cellular components, which can help to reduce the accumulation of senescent cells. Polyphenols have been shown to activate autophagy and promote the clearance of senescent cells.

Some polyphenols have also been shown to prevent senescence through their antioxidant and anti-inflammatory activity. These capacities may help to prevent the accumulation of senescent cells by reducing the damage and persistent inflammation exerted by ROS and pro-inflammatory cytokines that lead to senescence and by reducing the secretion of SASP factors, which can promote the senescence of neighboring cells. It is important to note that different polyphenols have different mechanisms of action, and not all of them have been thoroughly studied; additionally, the final outcomes can vary depending on the cell type and context.

In summary, among the various polyphenols that have been studied in relation to their senotherapeutic activity, quercetin, naringenin, and apigenin seem to be among the most effective. However, due to the complexity of the mechanisms involved under senescence and the variety of cell models used, it is difficult to determine which of these has clearly superior activity.

## 8. Conclusions

Cellular senescence is a permanent state of cell cycle arrest occurring in stressed cells that is driven by complex mechanisms, showing benefits and drawbacks. On the one hand, senescence growth arrest prevents tumorigenesis, limits fibrosis, and promotes tissue remodeling in development and wound healing. On the other hand, when accumulated, senescent cells promote persistent inflammation and oxidative stress and cell proliferation and invasion and may lead to aging and cancer progression. Senescence is also one of the causes of aging, and it is responsible for aging-related disorders. Therefore, senescence should be targeted in the development of innovative therapies because of its potential impact on several therapeutic areas.

Senescence is undoubtedly a highly heterogeneous phenomenon, and we suggest that a precise definition of the context will improve our understanding and allow for a reliable and meaningful comparison between different studies. Whether the process is beneficial or detrimental cannot be decided without considering the context. Senescence should be further studied to improve detection strategies; better understand how a resolution could occur and how it might be modulated; create populations that summarize the characteristics of all types of senescence (cell type, context, pathways, SASP, and biomarkers); and develop more targeted approaches for specific biomarkers.

The precise typification of senescence and the identification of its key features is controversial. According to the data analyzed in this review, the presence of a stable DNA damage response, the accumulation of SASP mediators, and the over-production of ROS appear to be the most representative hallmarks for senescent skin cells. Among the specific biomarkers that characterize senescent cells more precisely, the expression of the SA-β-gal enzyme seems to be the most reliable.

In an attempt to develop new anti-aging therapies, several plant polyphenols have been proposed to possess skin senotherapeutic activity based on several skin and animal models and human intervention studies. Among them, quercetin, naringenin, and apigenin seem to be the most effective. In addition to their antioxidant and anti-inflammatory activity, which prevents the increase in ROS and pro-inflammatory cytokines, the putative capacity of these compounds to eliminate or prevent the accumulation of senescent cells is proposed to take place through the inhibition of pro-survival signaling pathways, the activation of the autophagy process, and a decrease in the secretion of SASP factors. It should be also noted that the level of ROS is a key factor in the initiation and progression of the senescent process and in the accumulation of senescent cells and the SASP.

Although studies have provided evidence that senotherapeutics are effective at decreasing the number of senescent cells in humans, the short-term and long-term side effects of these therapies are largely unknown and necessitate further, more extensive investigation.

## Figures and Tables

**Table 1:** Main differences between cell senescence, quiescence, terminal differentiation, and T-cell exhaustion.

	Senescence	Quiescence	Terminal Differentiation	T-Cell Exhaustion	References
Type of cell cycle arrest	Generally irreversible	Reversible	Generally irreversible	Largely irreversible	[26,27,29,30]
Cause	Repetitive stimulation; DNA damage agents; stress signals	Signals of mitogen deprivation; contact inhibition	Genetically preprogramed	Continuous antigenic stimulation	[26,27,31,32]
Typical features	Large flat cells	Reduced cell size	n/a	n/a	[33,34]
	Cell cycle arrest driver: ↑ p16, p21, p53	CDK inhibitors: ↑ p21,27, 57	p21, p27, and p57	↑ p27, p15; ↓ cyclin E-Cdk2, Cdc25A	[26,27,31,35]
	↑ Macromolecular damage ↓ Telomere length, telomerase activity	Does not exhibit macromolecular damage	Does not exhibit macromolecular damage	↓ Telomere length, telomerase activity	[36,37,38]
	↑ SA-β-gal activity	Does not result in the upregulation of SA-β-gal activity	Does not result in the upregulation of SA-β-gal activity	Does not result in the upregulation of SA-β-gal activity	[39,40]
	n/a	n/a	n/a	↑ Inhibitory receptors: PD1, TIM3, LAG3, CTLA4, TIGIT	[41]
	↑ Glycolysis	↓↑ Glycolysis (depending on cell type)	n/a	↓ Glycolysis	[42,43,44]
Cytokine pattern	SASP, proinflammatory cytokines: ↑ IL-1, IL-6, IL-8, IFN-γ, TNF	n/a	n/a	↓ IL-2 ↓ TNF ↓ IFN-γ, β-chemokines	[45,46]
Epigenetic changes	↑ SAHF Abnormal DNA methylation	↑ H3K27me3 chromatin modifications; expression level of several histones is strongly reduced	↑ H3K9me3 and H3K27me3; reduced levels of global DNA methylation; enhancers are enriched for H3K27me3 and DNA methylation, which is associated with the lower expression of their target genes	Exhaustion-associated DNA methylation patterns	[37,47,48,49]

CDC25A: M-phase inducer phosphatase 1; CDK: cyclin-dependent kinase; CTLA-4: cytotoxic T-lymphocyte antigen 4; H3K27me3: trimethylation of lysine 27 on histone H3 protein; H3K9me3: trimethylation of lysine 9 on histone H3 protein; IFN-γ: interferon gamma; IL: interleukin; LAG-3: lymphocyte-activation gene 3; p16: cyclin-dependent kinase inhibitor 2A; p21: cyclin-dependent kinase inhibitor 1; p27: cyclin-dependent kinase inhibitor 1B; p53: cellular tumor antigen p53; p57: cyclin-dependent kinase inhibitor 1C; PD-1: programed cell death protein 1; SA-β-gal: senescence-associated beta-galactosidase; SAHF: senescence-associated heterochromatic foci; SASP: senescence-associated secretory phenotype; TIGIT: T-cell immunoreceptor with Ig and ITIM domains; TIM3: T-cell immunoglobulin and mucin domain-containing protein 3; TNF-α: tumor necrosis factor alpha; ↑: increase; ↓: decrease.

**Table 2:** Biomarkers of cellular senescence and general detection methods used to identify them.

Hallmarks of Senescence	Biomarker	Observation	Detection Method	References
Cell cycle arrest	p16/pRB axis	↑ p16 ↑ pRb ↓ Phospho-pRb	WB, IHC, IF	[56,115,116,117,118,119]
	p53/p21 axis	↑ p21 ↑ p53 ↑ Phospho-p53		
	Absence of proliferation	↓ Ki67 ↓ PCNA	IHC, IF	[24,120,121]
	Decrease in/absence of DNA synthesis	↓ BrdU, EdU	Staining incorporation, immunofluorescence	[122]
Metabolic adaptations	SA-β-gal	↑	NIR Fluorescence Enzymatic staining	[123,124,125]
	α-fucosidase	↑	Fluorescence Enzymatic staining	[64,126]
	Lipofuscin	↑	Dye incorporation (SSB, GL13) Fluorescence	[127,128]
Morphological changes	Wide and flattened cells High vacuolization	n/a	IF Scanning electron microscopy Light microscopy Flow cytometry	[129,130]
	Lamin B1	↓	qPCR, IF, WB	[131]
	Plasma membrane proteins	↑ ICAM-1, DEP1	Immunohistochemistry IF, WB, flow cytometry	[132]
Epigenetic alterations	SAHF	↑ PML bodies ↑ H3K9 methylation	IF	[133]
DNA damage	γH2AX 53BPI ATM ATR TIF	↑	IF	[89]
	Telomere shortening	↓	qPCR, FISH	[134,135]
Apoptosis resistance	Annexin V Cleaved caspases Cleaved PARP	↓/absent	IF IHC WB	[136,137,138,139,140]
	Blunt ends of double-stranded DNA breaks	-	TUNEL assay	[141]
Secretory phenotype	SASPs	↑ IL-1, IL-6, IL-8, ↑ TNF-α, GROα/β, ↑ MMP-1, MMP-3, MMP-9 ↑ IGFBPs ↑ SERPINs ↑ TIMPs	ELISA Immunofluorescence WB SASP-responsive alkaline phosphatase assay	[142,143,144,145]
ROS	O_2_, H_2_O_2_, O_2_^•−^, HO^•^	↑	Chemiluminescent oxygen detection, fluorometry, flow cytometry	[146,147]

γH2AX: phosphorylated histone H2AX; 53BPI: p53-binding protein; 8-OHdG: 8-hydroxydeoxyguanosine; ATM: ataxia-telangiectasia mutated; ATR: ATM and Rad3-related; BrdU: bromodeoxyuridine; DEP-1: density-enhanced phosphatase 1; EdU: 5-ethynyl-2′-deoxyuridine; FISH: fluorescence in situ hybridization; GRO: growth-regulated protein; ICAM-1: intercellular adhesion molecule 1; IF: immunofluorescence; IGFBPs: insulin-like growth factor binding proteins; IHC: immunohistochemistry; IL: interleukin; MMP: matrix metalloproteinase; NIR: near infrared; p16: cyclin-dependent kinase inhibitor 2A; p21: cyclin-dependent kinase inhibitor 1; p53: cellular tumor antigen p53; pRb: retinoblastoma protein 1; PARP: poly (ADP-ribose) polymerase; PCNA: proliferating cell nuclear antigen; PML: promyelocytic leukemia; ROS: reactive oxygen species; SA-β-gal: senescence-associated beta-galactosidase; SAHF: senescence-associated heterochromatin foci; SASP: senescence-associated secretory phenotype; SEM: scanning electron microscopy; SERPINs: serine protease inhibitors; TIMPs: tissue inhibitors of metalloproteinases; TIF: telomere dysfunction-induced foci; TUNEL: terminal deoxynucleotidyl transferase dUTP nick end labeling; WB: western blot; ↑: increase; ↓: decrease.

**Table 3:** Polyphenols with combined antioxidant capacity and skin senotherapeutic activity: the route of administration, research model used, aging inductor, and proposed mechanisms of action.

Polyphenol	Route of Administration	Aging Inductor	Research Model	Mechanism	Main Senotherapeutic Effects	Reference
Apigenin	In vitro	UVA and UVB	Human dermal fibroblasts	↓ ROS ↓ NF-kB pathway ↓ MAPK ↓ MMP-1	↑ Viability ↑ Collagen synthesis ↑ DNA repair	[148,168]
	Topical	UVA	Mice	↓ ROS ↓ NF-kB pathway ↓ MAPK ↓ MMP-1	↑ Dermal thickness ↑ Collagen deposition	[169]
Baicalin	In vitro	UVB	Human dermal fibroblasts, human skin samples	↓ ROS ↓ MMP-1, MMP-3 ↓ p16, p21, p53	↑ Collagen synthesis ↑ Viability ↓ DNA damage ↓ Apoptosis	[81]
	In vitro	UVC	Human keratinocytes	↓ ROS	↓ DNA damage	[170]
Ferulic acid	In vitro	UVA	Human dermal fibroblasts	↓ ROS ↑ SOD1 ↑ CAT ↓ p16 ↓ MMP-1, -3	↑ Proliferation and cell cycle ↑ ECM reconstruction	[171]
Fisetin	In vitro	Hydrogen peroxide	Human keratinocytes	↓ ROS ↓ NF-kB ↓ iNOS ↓ COX-2 ↓ IL-1β, -6, TNF-α	↓ SASP secretion ↑ Viability	[172]
	In vitro	UVB	Human dermal fibroblasts	↓ ROS ↓ MAPK/AP-1/MMP	↓ SASP secretion ↓ Collagen degradation	[173]
Gallic acid	In vitro	UVB	Human dermal fibroblasts	↓ ROS ↓ MMP-1 ↓ IL-6	↑ Procollagen type I	[174]
	Topical and oral	UVB	Mice	↑ TGF-β1 ↓ MMP-1 ↓ IL-6	↓ Wrinkle formation ↓ Skin dryness ↑ Procollagen type I ↑ Elastin	
Genistein	Topical	UVB	Mice	↓ ROS ↓ DNA pyrimidine dimer formation	↓ DNA damage	[175]
Luteolin	In vitro and topical	UVA	Human dermal fibroblasts, human keratinocytes, and human skin explants	↓ ROS ↓ MMP-1 ↓ IL-6, -20 ↓ p38/MAPK	↓ SASP secretion ↓ Collagen degradation ↓ Hyaluronic acid degradation	[176]
Naringenin	Intraperitoneal	UVB	Mice	↓ ROS ↓ MMP-9 ↓ TNF-α, IFN-γ ↓ IL-1β, -4, -5, -6, -12, -13, -17, -22, -23	↓ SASP secretion ↓ Inflammatory infiltrations	[177]
	Topical	UVB	Mice	↓ ROS ↓ IL-1β, -6, -10, TNF-α	↓ SASP secretion	[178]
Nectandrin B	In vitro	Cell passage ≥72	Human diploid fibroblasts	↓ ROS ↑ AMPK ↓ p16, p21, p27, p53 ↓ Cyclin D1 ↓ SA-β-gal ↓ Caveolin-1	↓ Senescence ↓ Apoptosis	[179]
Piceatannol	In vitro	UVB	Human keratinocytes	↓ ROS ↑ GSH ↓ NF-kB ↓ MMP-1	↓ Melanogenesis ↑ Collagen synthesis ↓ Photoaging	[180]
Quercetin	In vitro	Cell passage ≥17	Human dermal fibroblasts	↓ ROS ↑ SOD2, -3 ↑ CAT ↓ p16, p53	↓ Senescence ↑ Mitochondrial membrane potential	[181]

AMPK: AMP-activated protein kinase; AP-1: activator protein 1; CAT: catalase; ECM: extracellular matrix; GPx: glutathione peroxidase; GSH-Px: plasma glutathione peroxidase; HO-1: heme oxygenase 1; IFN-γ: interferon gamma; IL: interleukin; MAPK: mitogen-activated protein kinase; MCP-1: monocyte chemoattractant protein-1; MDA: malondialdehyde, COX-2: cyclooxygenase 2; MMP: matrix metalloproteinase; NF-kB: nuclear factor kappa B; nHDF: normal human dermal fibroblasts; nPC12: neuronally differentiated phenchromocytoma cells; p16: cyclin-dependent kinase inhibitor 2A; p21: cyclin-dependent kinase inhibitor 1; p27: cyclin-dependent kinase inhibitor 1B; p38/MAPK: mitogen-activated protein kinase p38; ROS: reactive oxygen species; SA-β-gal: senescence-associated beta-galactosidase; SASP: senescence-associated secretory phenotype; SOD: superoxide dismutase; TGF-β1: transforming growth factor beta 1; TNF-α: tumor necrosis factor alpha; UVA: ultraviolet A; UVB: ultraviolet B; UVC: ultraviolet C; ↑: increase; ↓: decrease.

## Data Availability

The data presented in this study are available in the article.
